# An improved and high-throughput mpox virus microneutralization assay

**DOI:** 10.1016/j.bsheal.2026.01.003

**Published:** 2026-01-30

**Authors:** Danyang Li, Zuoyuan Du, Qiao Zhang, Rui Song, Lan Chen, He Huang, Jianwei Wang, Li Guo, Lili Ren

**Affiliations:** a National Health Commission Key Laboratory of Systems Biology of Pathogens and Christophe Mérieux Laboratory, Chinese Academy of Medical Sciences & Peking Union Medical College National Institute of Pathogen Biology, Beijing 102629, China; b Beijing Ditan Hospital Capital Medical University, Beijing 100015, China; c Beijing Key Laboratory of Surveillance, Early Warning and Pathogen Research on Emerging Infectious Diseases, Beijing Center for Disease Prevention and Control, Beijing 100013, China; d Key Laboratory of Respiratory Disease Pathogenomics, Chinese Academy of Medical Sciences & Peking Union Medical College, Beijing 100730, China; e Key Laboratory of Pathogen Infection Prevention and Control of Ministry of Education, State Key Laboratory of Respiratory Health and Multimorbidity, National Institute of Pathogen Biology, Chinese Academy of Medical Sciences & Peking Union Medical College, Beijing 102629, China

**Keywords:** Mpox virus (MPXV), Microneutralization assay, Monoclonal antibodies (mAbs), A35 protein

## Abstract

Since the 2022 global mpox outbreak, the lack of specific antibodies for mpox virus (MPXV) detection has hindered precise immunoassays due to cross-reactivity among *Orthopoxviruses* (OPXVs). This study developed and characterized two monoclonal antibodies (mAbs), CML01 and CML02, targeting the MPXV A35 protein, a conserved surface antigen of extracellular virions. Cross-reactivity assessments via enzyme-linked immunosorbent assay (ELISA), western blot, and indirect immunofluorescence assays (IFA) confirmed that both mAbs bound exclusively to MPXV A35, showing no reactivity with homologous proteins from cowpox (A34), vaccinia (A33), or variola viruses (A36) despite 92.3 %–96.1 % sequence homology. IFA showed recognition of MPXV-infected cells with half-maximal effective concentrations (EC_50_) of 0.15 and 0.17 μg/mL, respectively. Notably, an IFA-based microneutralization assay using the mAb CML02 exhibited strong correlation (*r* = 0.93, *P* < 0.0001) with the traditional plaque reduction neutralization test (PRNT) while enabling higher throughput. Plasma from convalescent mpox patients validated the assay’s utility in testing neutralizing antibody titers. These mAbs address critical gaps in MPXV-specific immunological testing by virtue of their high specificity, which prevents cross-reactivity with other OPXVs and eliminates interference from immunity induced by smallpox vaccination. This work underscores A35 as a key epitope for MPXV-specific immunity and provides essential tools for combating the ongoing mpox threat.

## Highlights

• **Scientific question:** The detection of specific antibodies for mpox virus (MPXV) has posed significant challenges owing to cross-reactivity among *Orthopoxviruses*. Moreover, high-throughput assays for detecting MPXV–specific neutralizing antibodies remain lacking.
• **Evidence before this study:** MPXV shares > 90 % nucleotide sequence homology with cowpox virus (CPXV), variola virus (VARV), and vaccinia virus (VACV), leading to significant antigenic cross-reactivity. Current MPXV detection and neutralization assays mainly rely on polyclonal antibodies raised against VACV, which lack specificity for MPXV, thereby complicating the differentiation between MPXV infection and infections caused by other OPXVs or immunity induced by smallpox vaccination. Conventional plaque reduction neutralization test (PRNT), while widely used for neutralizing antibody (NAb) against MPXV, suffers from low throughput and technical complexity.
• **New findings:** The study successfully developed and characterized two monoclonal antibodies (mAbs), CML01 and CML02, which specifically target the MPXV A35 protein. Despite high sequence homology, these mAbs exhibited high specificity with no cross-reactivity with homologous proteins from CPXV, VARV, and VACV. A microneutralization assay based on indirect immunofluorescence assay (IFA) utilizing mAb CML02 showed a strong correlation (*r* = 0.93, *P* < 0.0001) with traditional PRNT, thereby enabling higher-throughput detection of neutralizing antibodies against MPXV with specificity.
• **Significance of the study:** The MPXV-specific monoclonal antibodies eliminate cross-reactivity with other orthopoxviruses, thereby enabling precise differentiation of MPXV infection from immune responses elicited by other OPXVs or prior smallpox vaccination. The development of a high-throughput neutralization assay based on MPXV-specific mAb streamlines NAb detection, facilitating rapid evaluation of vaccine efficacy and population immunity during outbreaks.

## 1 Introduction

Since May 2022, mpox has spread globally, primarily through person-to-person transmission via close contact with individuals infected with the mpox virus (MPXV) [1]. Mpox was declared a Public Health Emergency of International Concern (PHEIC) on July 23, 2022 [2], and August 14, 2024 [3]. The declarations underscore the extensive research into MPXV to enhance our understanding of the virus and improve disease control strategies. Immunological assays, such as enzyme-linked immunosorbent assay (ELISA), western blot, immunofluorescence assay (IFA), microneutralization test, and immuno-colloidal gold technique, rely on the use of specific antibodies. These assays are widely employed in studies of viral transmission, epidemiological characteristics, pathogenesis, immunology, clinical diagnosis, and the development of control strategies. The MPXV, cowpox virus (CPXV), variola virus (VARV), and vaccinia virus (VACV), members of the *Orthopoxvirus* (OPXV), are capable of causing infections in humans [4]. MPXV, CPXV, VARV, and VACV exhibit greater than 90 % nucleotide identity [5], leading to these viruses sharing many genetic and antigenic features. Therefore, the specificity of antibodies to MPXV directly determines the efficiency and reliability of the immunological assays.

Anti-OPXV antibodies have shown significant cross-reactivity with a broad range of OPXV species, including MPXV, CPXV, VARV, and VACV [4]. Research on antibodies targeting the MPXV has mainly focused on broad-spectrum neutralizing antibodies (NAbs) capable of recognizing not only the MPXV but also other OPXVs, such as VACV [6], [7]. Recently, Kurosawa et al. developed a monoclonal antibody (mAb) against the MPXV A5 protein and established an antigen-detection-based rapid diagnostic test for MPXV. No cross-reactivity with VACV was observed at concentrations below 1.76 × 10^4^ plaque forming unit (PFU) / test; However, cross-reactivity was detected at higher VACV titers [8]. Two long-lasting human monoclonal antibodies (mAbs), isolated from MPXV-specific memory B cells, exhibit cross-reactivity with the VACV A33 and MPXV A35 antigens in immunoblot and IFA [9]. M. Hubert et al. [10] conducted an MPXV neutralization assay using polyclonal antibodies against VACV. Thus, highly specific assays for the detection of MPXV infection remain limited. Although several commercially available polyclonal antibodies targeting MPXV have been used for ELISA and immunoblotting, they are generally unsuitable for IFA. NAbs are widely recognized as the primary serological marker of protection. Nevertheless, the conventional plaque reduction neutralization test (PRNT), which quantifies the reduction in plaque-forming units due to the presence of MPXV-specific antibodies, remains one of the most commonly used methods. However, the detection throughput of the PRNT is relatively low. D. Li et al. [11] performed a focus reduction neutralization test (FRNT) using anti-VACV polyclonal antibodies to detect NAbs against MPXV. However, the antibody lacked specificity to MPXV. Consequently, it is challenging to differentiate MPXV infection from immune responses elicited by other OPXVs or prior smallpox vaccination.

The A35 protein of MPXV, akin to the A33 protein of VACV, the A36 protein of VARV, as well as A34 of CPXV, is highly conserved among MPXV, CPXV, VARV, and VACV. Serving as surface proteins of the extracellular enveloped virion (EEV), the A35 protein is a potential target for vaccine development [12], [13], detection reagents [14], and antiviral drugs [15]. In this study, we produced and characterized two mAbs targeting the A35 protein of MPXV. These mAbs showed specificity for MPXV and can be utilized in immunoassays for the detection of MPXV, as well as for performing high-throughput neutralization assays.

## 2 Materials and methods

### 2.1 Plasma specimens and virus

Plasma samples were collected from convalescent patients with polymerase chain reaction (PCR)-confirmed MPXV infection and from individuals born before 1980 at Beijing Ditan Hospital Capital Medical University in Beijing, China.

The MPXV (IPBCAMS-MP05-1/2023) strain was isolated from skin lesions of an mpox patient in a biosafety level 3 (BSL-3) laboratory of the National Institute of Pathogen Biology, Chinese Academy of Medical Sciences.

### 2.2 Expression and purification of recombinant A35 protein

The *A35R* gene of MPXV (Clade II; GenBank accession number AF380138.1), the *A34R* gene of CPXV (GenBank accession number LT896731.1), the *A33R* gene of VACV (GenBank accession number NC_006998.1), and the *A36R* gene of VARV (GenBank accession number NC_001611.1) were fused with a 6 × His tag and cloned into the pCAGGS vector, respectively. The recombinant plasmids were transfected into Expi293F cells for 5 days to produce the fusion proteins. The fusion proteins were subsequently purified using Ni-NTA His-Tag Purification Agarose (MedChemExpress) according to the manufacturer's instructions. The A35 protein of Clade I MPXV was purchased from ACROBiosystems, Beijing, China.

### 2.3 Preparation of mAbs against the A35 protein of MPXV

MAbs generation was performed at Universal Biology Inc. (Hefei, China) in compliance with approved animal ethics protocols (Approval No.23-Nov-2). Six- to eight-week-old female BALB/c mice were inoculated subcutaneously with the purified recombinant the A35 protein of MPXV, mixed with complete or incomplete Freund's adjuvant, at two-week intervals. After ELISA assessment, mice with the highest antibody titers were selected. Splenocytes from immunized mice were fused with SP2/0 myeloma cells in the presence of polyethylene glycol (PEG) solution [Sigma-Aldrich, St. Louis, MO, the United States of America (USA)]. The fused cells were cultured in 96-well plates using hypoxanthine-aminopterin-thymidine (HAT) selection medium (Sigma-Aldrich). After 7–10 days, positive hybridoma clones were identified by ELISA. Hybridoma supernatants were collected, and antibody responses against the A35 protein of MPXV were analyzed using ELISA. Following three rounds of subcloning, single-cell clones exhibiting higher optical density at 450 nm (OD_450_) values were expanded and cultured. The mAbs in hybridoma supernatants were purified using protein L (GenScript, Nanjing, China).

### 2.4 ELISA

100 ng/well of the A35 protein of MPXV, A34 protein of CPXV, A33 protein of VACV, and A36 protein of VARV were coated in a high-binding 96-well plate (Corning, NY, USA), respectively, using carbonate buffer. After incubation overnight at 4 °C, the plates were incubated with blocking buffer [1 × phosphate- buffered saline (PBS) supplemented with 2 % bovine serum albumin (BSA), Sigma-Aldrich] for 2 h at 37 °C. The anti-MPXV-A35 mAb was initially diluted at 10 μg/mL and subjected to three-fold serial dilutions. The diluted mAbs were then added to the plates and incubated for 1 h at 37 °C. Following washing, peroxidase-conjugated rabbit anti-mouse specific IgG (Sigma-Aldrich) was added to the plates. After 1 h of incubation at 37 °C, the plates were washed and developed with 100 μL of 3,3′,5,5′-tetramethylbenzidine (TMB) two-component substrate solution (Solarbio). The reaction was stopped by adding 50 μL of stop buffer (Solarbio). The OD_450_ was determined with EnSight (Revvity, Waltham, MA). EC_50_ values were calculated from dose-response curves fitted to the OD_450_ values at varying monoclonal antibody concentrations using GraphPad Prism. The IgG subclasses of the mAbs were identified using the Mouse Monoclonal Antibody Subclass Identification ELISA Kit (Proteintech, Wuhan, China) in accordance with the manufacturer's instructions.

### 2.5 Western blot

Purified A35 protein of MPXV, A34R protein of CPXV, A33R protein of VACV, and A36 protein of VARV, as well as lysates from Vero cells infected with MPXV or VACV, were separated by 15 % sodium dodecyl sulphate polyacrylamide gel electrophoresis (SDS-PAGE) gels and transferred to a nitrocellulose membrane (Pall, Port Washington, NY, USA). The anti-MPXV A35 mAbs were applied to probe MPXV, CPXV, VACV, and VARV, respectively. IRDye 800CW goat anti-mouse IgG secondary antibody was used at a dilution of 10,000 (Li-Cor, Lincoln, NE, USA). The membranes were scanned using the Odyssey Infrared Imaging System (Li-Cor). Vaccinia virus polyclonal antibody targeting vaccinia virus B5 and A33 protein (Invitrogen, Cat no. PA1-7258) was used as a positive control.

### 2.6 Indirect IFA

Vero cells were infected with MPXV in a BSL-3 laboratory or infected with VACV in a BSL-2 laboratory at a multiplicity of infection (MOI) of 0.1 in 96-well plates (Costar, NY, USA) for 48 h. The cells were then fixed with 4 % formaldehyde, permeabilized with 0.1 % Triton X-100, and blocked with 5 % BSA. The anti-MPXV A35 mAbs were initially diluted to 2 μg/mL and subjected to two-fold serial dilutions, and incubated for 1 h at room temperature. Alexa Fluor 488-labeled anti-mouse IgG (Life Technologies, Eugene, OR, USA) was used as the secondary antibody at a dilution of 1:1,000. After incubation for 1 h, nuclei were stained with 4′,6-diamidino-2-phenylindole (DAPI) (Sigma-Aldrich). Fluorescence images were acquired using an Operetta High Content Screening system (PerkinElmer, Waltham, MA, USA). The number of nuclei and MPXV-positive cells was quantified using Harmony software (PerkinElmer). EC_50_ values were calculated from dose-response curves fitted to the percentage of MPXV-positive cells at varying monoclonal antibody concentrations using GraphPad Prism. A vaccinia virus polyclonal antibody targeting vaccinia virus B5 and A33 protein (Invitrogen) was used as a positive control.

### 2.7 Microneutralization assay against MPXV based on IFA

NAbs against MPXV in plasma were assessed in Vero cells (ATCC, Manassas, VA, CCL-81) infected with MPXV based on IFA. Vero cells were plated at a density of 1.5 × 10^4^ cells per well in a 96-well plate. Plasma samples were heat-inactivated at 56 °C for 30 min and then subjected to two-fold serial dilutions ranging from 1:4 to 1:512. These serially diluted plasma samples were preincubated with MPXV at an MOI of 0.1 for 1 h at 37 °C in the presence of 10 % guinea pig serum (Solarbio) serving as a source of complement. Subsequently, the virus-plasma mixtures were then added to Vero cell monolayers in 96-well plates and incubated for 2 h. Following this incubation period, the virus-plasma mixtures were removed, and the wells were washed twice with PBS. Subsequently, 100 μL of fresh Dulbecco's modified Eagle medium (DMEM) supplemented with 2 % fetal bovine serum (FBS) was added to each well. Forty-eight hours later, cells were fixed with 4% formaldehyde for 30 min, permeabilized with 0.1 % Triton X-100, and blocked with 5 % BSA. The cells were then immunostained for MPXV antigens using mAb CML02 at a concentration of 0.125 μg/mL, followed by an Alexa Fluor 488-conjugated anti-mouse IgG secondary antibody. Nuclei were stained with DAPI. Fluorescence images were acquired using an Operetta High Content Screening system. The MPXV-positive cells and the number of nuclei were quantified using the Harmony software. The neutralization titers were calculated using a reconstructed curve with the percentage of neutralization at the different plasma dilutions using GraphPad Prism. The NT_50_ was defined as the highest plasma dilution resulting in more than 50 % reduction in MPXV-positive signal compared to the “no plasma” control.

### 2.8 PRNT test

The plasma samples were also subjected to the PRNT to determine the neutralization titers as previously described [16]. Briefly, serial four-fold dilutions of plasma samples (starting at 1:10) were pre-incubated with MPXV at 75 PFU in the presence of 10 % guinea pig serum (Solarbio) serving as a source of complement. After 1 h of incubation at 37 °C, the virus–plasma mixture was added to Vero cells seeded in 12-well plates (Costar, NY, USA). Following a 2 h incubation, the inoculum was removed, and 1 mL of fresh overlay medium (DMEM supplemented with 2 % FBS and 1 % methyl cellulose) was added to each well. At three days post-infection, the plates were stained with crystal violet, and plaque numbers were quantified. Each plasma dilution was tested in duplicate wells. The neutralization titers were calculated using a dose-response curve fitted to the percentage of neutralization at different plasma dilutions using GraphPad Prism. Antibody titers were defined as the highest plasma dilution yielding greater than 50 % reduction in viral plaques (PRNT_50_). A titer of 1:10 was considered the threshold for a positive neutralizing antibody response. When neutralizing antibody levels were below the limit of detection, the titer was assigned a value of 1:5.

### 2.9 Amino acid sequence comparison

The A35 protein sequences of MPXV, the A34 protein sequences of CPXV, the A33 protein sequences of VACV, and the A36 protein sequences of VARV were aligned using the ClustalW program in MEGA v12 software.

### 2.10 Statistical analysis

The neutralizing antibody titers determined by IFA and PRNT were log2-transformed. The Shapiro-Wilk test was used to assess the normality of the transformed data. Spearman correlation analysis was performed on the log2-transformed neutralizing antibody titers determined by IFA and PRNT. All statistical analyses were performed using GraphPad Prism v10.5.

## 3 Results

### 3.1 Sequence homology comparison and protein purification

**Fig. 1:**
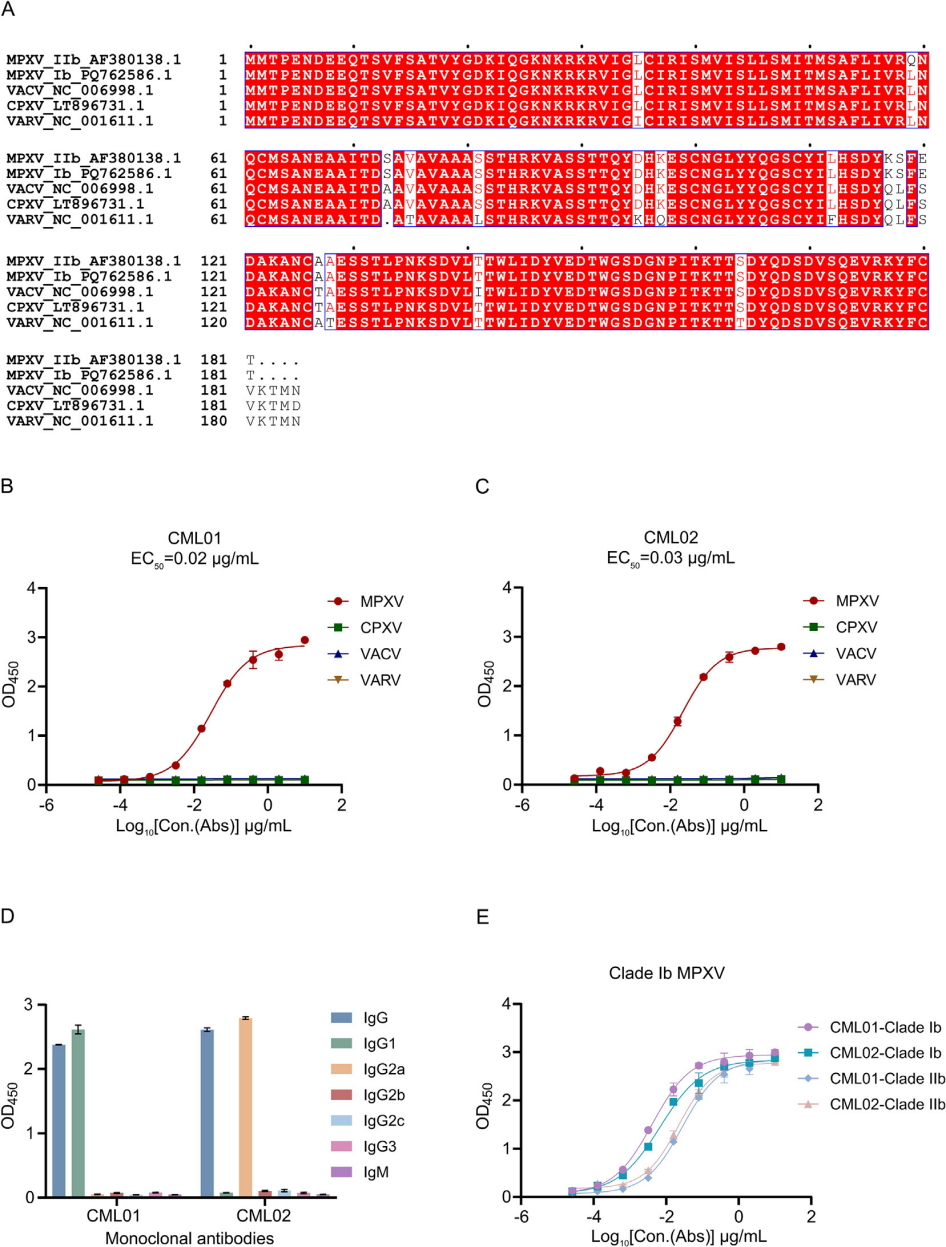
Identification of monoclonal antibodies specific to MPXV A35 protein. A) Amino acid sequence comparison of the clade IIb MPXV A35 protein with homologous proteins from Clade Ib MPXV, VACV, CPXV, and VARV. B–C) The reactivities of monoclonal antibodies CML01 (B) and CML02 (C) to Clade II MPXV A35, CPXV A34, VACV A33, and VARV A36 proteins were evaluated using ELISA. D) IgG subclasses of CML01 and CML02 were identified using ELISA. E) The reactivities of monoclonal antibodies CML01 and CML02 to the A35 protein of Clade Ib MPXV were evaluated using ELISA. NT_50_ was defined as the highest plasma dilution resulting in more than 50 % reduction in MPXV-positive signal compared to the “no plasma” control. Abbreviations: EC_50_, the half maximal effective concentration; MPXV, mpox virus; CPXV, cowpox virus; VACV, vaccinia virus; VARV, variola virus; ELISA, enzyme-linked immunosorbent assay; Abs, antibodies; Con, concentration.

Pairwise analysis showed the A35 protein of Clade IIb MPXV had 96.1 %, 95.6 %, and 92.3 % amino acid (aa) sequence homology with the corresponding proteins of CPXV, VACV, and VARV, respectively (Table 1, Fig. 1A). The A35 proteins of Clade IIb and Clade Ib MPXV share 99.8 % and 99.4 % nucleotide and amino acid sequence identity, respectively. The results suggest that these viruses exhibit a high degree of sequence homology. The *A35R* gene of MPXV, together with its homologous counterparts in CPXV, VACV, and VARV, was cloned into the pCAGGS vector and expressed using the Expi293F expression system. The proteins were purified by Ni-affinity chromatography, yielding a purity of over 90 %.

**Table 1:** Analysis of nucleotide and amino acid sequence homology of A35 among MPXV, CPXV, VACV, and VARV.

Virus	Nucleotide, % (amino acid, %)				
	MPXV (IIb)	MPXV (Ib)	CPXV	VACV	VARV
MPXV (IIb)	100.0 (100.0)				
MPXV (Ib)	99.8 (99.4)	100.0 (100.0)			
CPXV	96.9 (96.1)	97.1 (96.7)	100.0 (100.0)		
VACV	96.5 (95.6)	96.7 (96.1)	99.1 (98.9)	100.0 (100.0)	
VARV	92.9 (92.3)	93.0 (92.8)	95.9 (94.6)	96.0 (94.6)	100.0 (100.0)

Data outside parentheses indicate nucleotide sequence homology, while data within parentheses indicate amino acid sequence homology. Abbreviations: MPXV, mpox virus; CPXV, cowpox virus; VACV, vaccinia virus; VARV, variola virus.

### 3.2 Generation and characterization of mAbs against MPXV

Two anti-A35 mAbs, CML01 and CML02, were generated by fusing Sp2/0 myeloma cells with splenocytes from mice immunized with the A35 protein of Clade IIb MPXV. Specificity of the mAbs was initially evaluated using ELISA, with A35 protein of MPXV, the A34 protein of CPXV, the A33 protein of VACV, and the A36 protein of VARV as coating antigen. The results showed that the mAbs CML01 and CML02 showed specificity for MPXV, with EC_50_ values of 0.02 μg/mL (Fig. 1B) and 0.03 μg/mL (Fig. 1C) for ELISA, respectively. The subclasses of the two mAbs were identified as IgG1 for CML01 and IgG2a for CML02 (Fig. 1D), respectively, through ELISA analysis. Additionally, their light chains were confirmed to be κ chains based on sequence analysis (data not shown). These results indicate that the reactivity of anti-A35 mAbs CML01 and CML02 was specifically limited to MPXV.

To evaluate the reactivities of mAbs with Clade Ib MPXV strain, an ELISA was conducted using the A35 protein of Clade Ib MPXV as the coating antigen. The results showed that the anti-A35 mAbs CML01 and CML02 exhibited a similar reactivity pattern with Clade Ib MPXV compared to that observed with Clade II MPXV (Fig. 1E), which suggests that CML01 and CML02 can react with both Clade I and Clade II MPXV.

### 3.3 Immunoassays using the mAbs

**Fig. 2:**
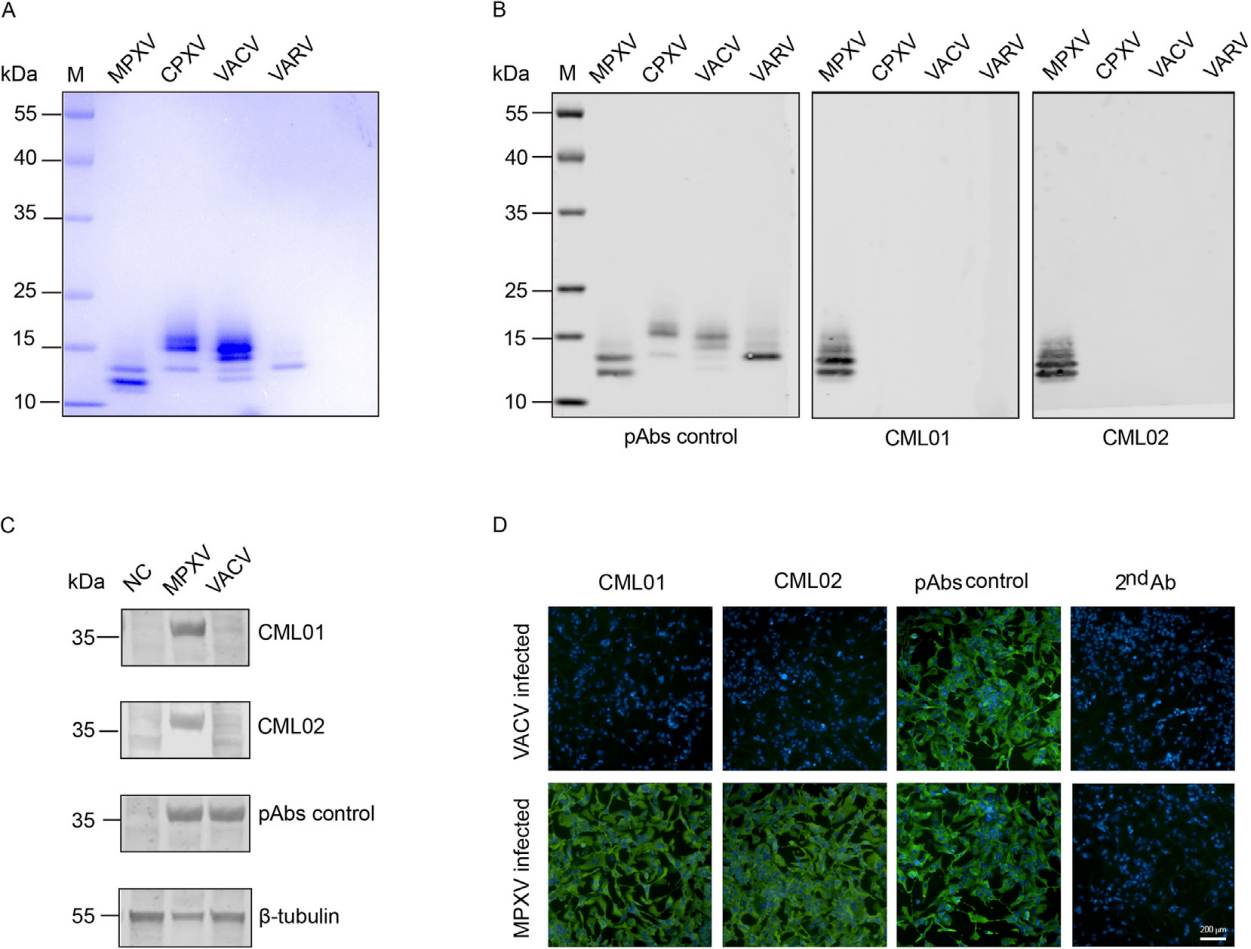
Characterization of monoclonal antibodies specific to MPXV-A35. A) SDS-PAGE analysis of purified MPXV A35 protein and its homologous proteins. B) The reactivities of mAbs CML01 and CML02 against the recombinant proteins of clade II MPXV A35, CPXV A34, VACV A33, and VARV A36 were evaluated by western blot. C) The reactivities of mAbs CML01 and CML02 against lysates from MPXV- or VACV-infected Vero cells were assessed by western blot. D) The reactivities of mAbs CML01 and CML02 with Vero cells infected with MPXV or VACV were assessed by indirect immunofluorescence assay. In B to D, a pAb targeting vaccinia virus B5 and A33 proteins (Invitrogen) was used as a positive control. Abbreviations: MPXV, mpox virus; CPXV, cowpox virus; VACV, vaccinia virus; VARV, variola virus; ELISA, enzyme-linked immunosorbent assay; mAbs, monoclonal antibodies; pAb, polyclonal antibody; NC, negative control; M, marker.

To determine the suitability of the mAbs CML01 and CML02 for immunoassays targeting MPXV, we evaluated their performance using Western blot and IFA. The purified A35 protein (Fig. 2A) was first subjected to SDS-PAGE, followed by Western blot analysis using the two monoclonal antibodies. The results showed that mAbs CML01 and CML02 specifically recognized the A35 protein of MPXV, with no cross-reactivity observed against the homologous proteins from CPXV, VACV, or VARV (Fig. 2B). In contrast, the polyclonal antibody raised against vaccinia virus was found to recognize A35 recombinant proteins across all tested orthopoxviruses, including MPXV, CPXV, VACV, and VARV (Fig. 2B). Furthermore, 40 μg of lysate from MPXV- or VACV-infected Vero cells was used as antigen to perform Western blot assays. The results showed that mAbs CML01 and CML02 specifically reacted with native conformation A35 protein in MPXV-infected Vero cells, with no cross-reactivity observed in VACV-infected Vero cells (Fig. 2C). Next, we conducted an IFA to detect MPXV- or VACV-infected Vero cells using mAbs CML01 and CML02. Consistent with the Western blot results, CML01 and CML02 specifically recognized MPXV-infected Vero cells, but not VACV-infected Vero cells (Fig. 2D). Collectively, these findings indicate that CML01 and CML02 are specific mAbs against MPXV.

**Fig. 3:**
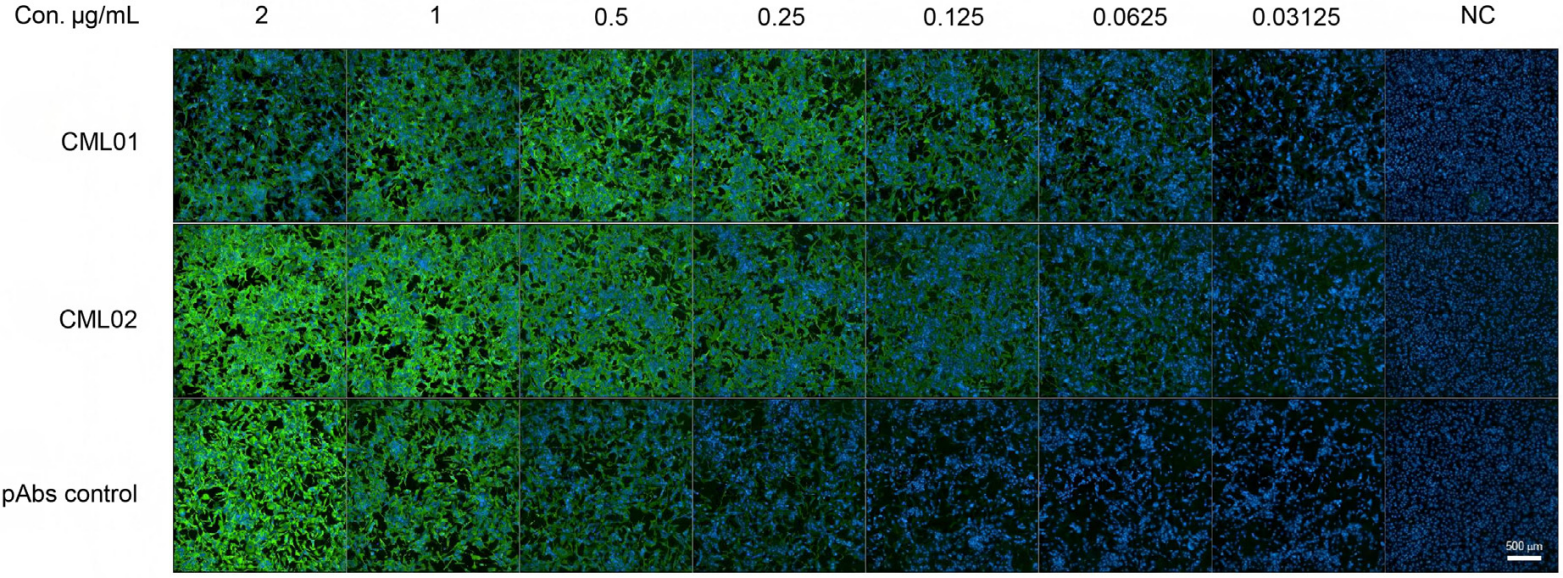
The reactivities of monoclonal antibodies CML01 and CML02 to MPXV-A35 using indirect immunofluorescence assay. The reactivities of mAbs CML01 and CML02 at various concentrations against Vero cells infected with MPXV were assessed using an indirect immunofluorescence assay. A polyclonal antibody targeting Vaccinia virus B5 and A33 proteins (Invitrogen) was used as the positive control. Uninfected Vero cells were used as a negative control, tested with the highest concentration of CML01 and CML02 (2 μg/mL). Abbreviations: MPXV, mpox virus; pAbs, polyclonal antibodies; NC, negative control; Con, concentration; mAbs, monoclonal antibodies.

The EC_50_ values of mAbs for IFA were determined using Vero cells infected with MPXV and serial dilutions of CML01 and CML02. CML01 and CML02 exhibited EC_50_ values of 0.15 μg/mL and 0.17 μg/mL, respectively, when tested against MPXV. As shown in Fig. 3, both mAbs CML01 and CML02 displayed strong positive fluorescence signals at 0.125 μg/mL weak. However, detectable signals still observed at 0.0625 μg/mL. In contrast, polyclonal antibody against VACV exhibited positive fluorescence at a higher concentration of 1 μg/mL. Neither of the two monoclonal antibodies, CML01 and CML02, showed reactivity with uninfected Vero cells even at the highest concentration of 2 μg/mL (Fig. 3). These results suggest that mAbs CML01 and CML02 possess high specificity and sensitivity, supporting their potential application in the development of immunoassays for MPXV detection.

### 3.4 Application of mAb CML02 in high-throughput microneutralization assays

**Fig. 4:**
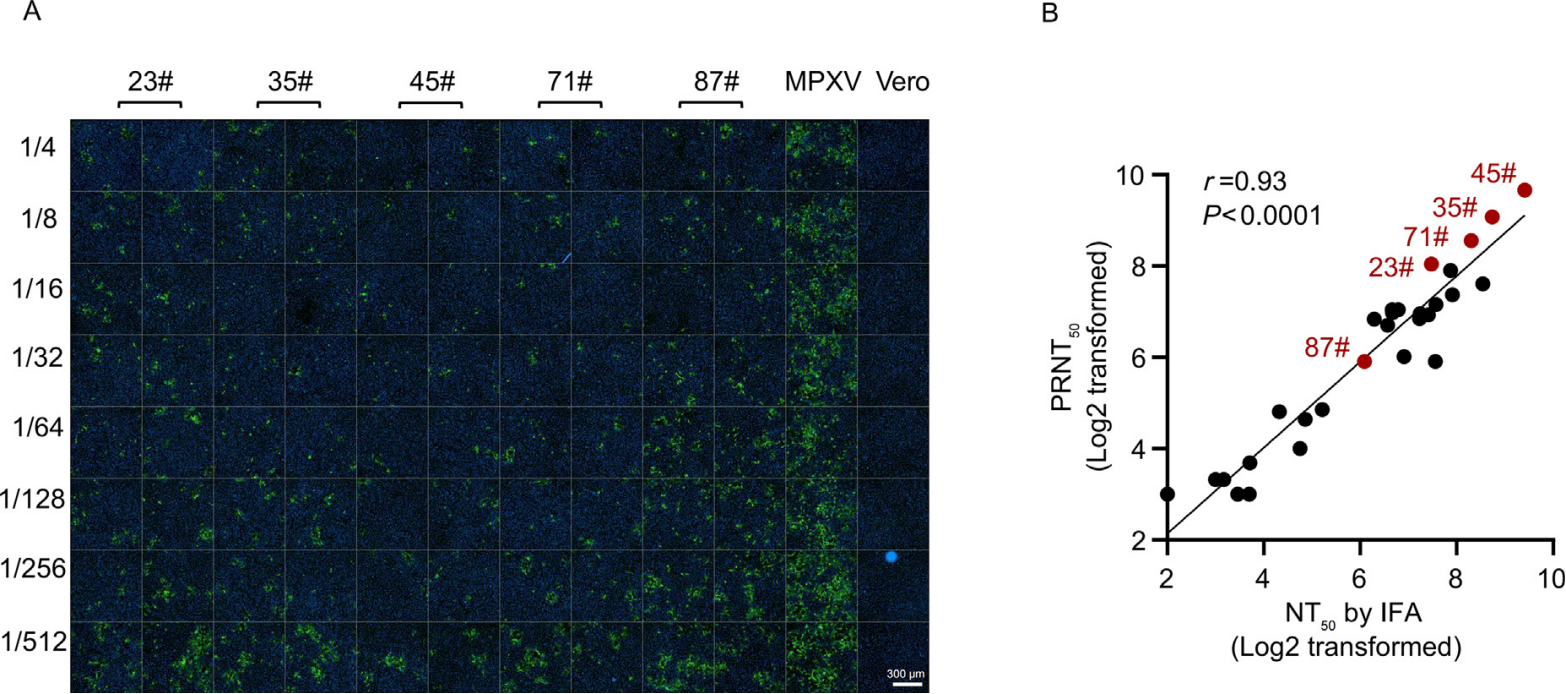
Application of mAb CML02 in microneutralization assays. A) Neutralizing antibodies were assessed on Vero cells infected with MPXV/plasma mixtures, then fixed with 4% formaldehyde and subjected to an IFA using mAb CML02. B) The correlation of neutralizing antibody titers between microneutralization assays based on IFA and PRNT using mAb CML02. The correlation analysis was conducted using Spearman correlation, based on the log2-transformed neutralizing antibody titers as determined by IFA and PRNT. NT_50_ was defined as the highest plasma dilution resulting in more than 50 % reduction in MPXV-positive signal compared to the “no plasma” control. Abbreviations: MPXV, mpox virus; IFA, indirect immunofluorescence assay; PRNT, plaque reduction neutralization test; mAbs, monoclonal antibodies.

We selected mAb CML02 to validate its application in microneutralization assays. Plasma samples from thirty convalescent Mpox patients and individuals born before 1980 were serially diluted and incubated with MPXV. The plasma/MPXV mixture was used to infect Vero cells for 2 h, after which the inoculum was removed. After 48 h post-infection, the Vero cells were fixed and subjected to IFA using mAb CML02 as the primary antibody to detect A35 protein expression. As shown in Fig. 4A, the expression of A35 increased with higher plasma dilution. The neutralizing titers (NT_50_) values of plasmas were determined based on immunofluorescence readouts. Fig. 4A shows the NT_50_ of five recovered patients, with values of 178, 426, 680, 316, and 68, respectively. The NAbs in all the plasma samples were evaluated by traditional PRNT concurrently. The PRNT_50_ values were 264, 539, 810, 376, and 60 for the five samples, respectively. A strong correlation was observed between NT_50_ values obtained by IFA and those derived from PRNT (*r* = 0.93, *P* < 0.0001; Fig. 4B). Therefore, the anti-A35 mAb CML02 provides a valuable tool for high-throughput microneutralization assays.

## 4 Discussion

In this study, we developed two mAbs targeting the A35 protein of MPXV. These mAbs showed specificity for MPXV, but not for CPXV, VARV, and VACV. They can be utilized in immunoassays for the detection of MPXV, including ELISA, IFA, and immunoblotting assay, as well as in high-throughput neutralization tests.

The increasing incidence of MPXV infections underscores the urgent need for the development of rapid and reliable immunological analysis methods. mAbs have been extensively utilized in the diagnosis and treatment of a wide range of diseases, owing to their high specificity, potent targeting capabilities, low toxicity, and minimal side effects [17]. It has been reported that an antigen capture ELISA using anti-VACV A27 mAb can detect OPXV isolates, including MPXV [18]. However, the broad-spectrum reactive mAbs are unable to differentiate MPXV from other OPXVs. In this study, we showed that an immunological test using anti-MPXV A35 mAbs can detect samples containing the A35 protein as well as Vero cells infected with MPXV, suggesting its potential application in diagnosing MPXV infection. Due to the unavailability of CPXV and VARV, cross-reactivity data at the infection or cellular level are currently limited to VACV. Conclusions regarding CPXV and VARV are therefore primarily based on recombinant protein assays.

One of the major challenges in MPXV research is the requirement for a BSL-3 facility when handling replication-competent virus, particularly for neutralization tests. The NAbs test for MPXV was usually conducted using the PRNT, with read-out based on plaques formed by the lysis of infected cells in a confluent cell monolayer [16], [19], [20]. However, this method is suitable only for small sample sizes. The development of a relatively simple, high-throughput assay would facilitate the assessment of NAbs in patients infected with MPXV and support the advancement of Mpox vaccine development. This is especially critical considering the growing number of MPXV vaccine candidates currently under investigation [21]. In this study, we developed a neutralization method that involves incubating virus/plasma mixtures with Vero cells in 96-well plates at a BSL-3 laboratory, followed by fixing the cells with formaldehyde and performing an IFA using anti-MPXV-specific mAb at BSL-2 laboratory. This method enables the high-throughput detection of specific NAbs against MPXV.

Specifically, our experimental data indicate that mAb CML01 and CML02 exhibit comparable efficacy in Western blot and IFA. However, CML02 demonstrates superior performance in IFA-based neutralization experiments, enabling reliable signal detection at a lower concentration (0.125 μg/mL) than CML01 (0.25 μg/mL). This enhanced sensitivity and efficiency meet the stringent requirements for reagent optimization in high-throughput screening platforms. The robustness and reproducibility of results obtained with CML02 across multiple experimental conditions support its selection as the preferred reagent.

The high sequence similarity among *Orthopoxviruses* (> 90%) poses a challenge in developing MPXV-specific mAbs for detection and neutralization assays. MPXV consists of two distinct genomic clades: clade I (formerly known as the Congo Basin clade), and clade II (previously called the West African clade). Notably, infections caused by clade I exhibit a higher case fatality rate compared to those caused by clade II [22], [23]. The mpox cases reported in China predominantly belong to clade IIb [24], [25]. However, a case of clade Ib MPXV infection has recently been reported [26]. In this study, the anti-MPXV-specific mAbs CML01 and CML02 were found to recognize both clade I and clade II with high specificity, enabling direct detection of MPXV infection and the establishment of high-throughput MPXV microneutralization assays.

## 5 Conclusions

In summary, this study developed two highly specific monoclonal antibodies (CML01 and CML02) targeting the MPXV A35 protein, enabling precise detection and differentiation of MPXV infection from other orthopoxviruses. The mAbs facilitated a high-throughput microneutralization assay, improving vaccine evaluation and outbreak response.

## Ethics statement

The study was approved by the Institutional Review Boards of Beijing Ditan Hospital Capital Medical University (2023–025). Written informed consent was obtained from all subjects before inclusion.
